# Leveraging ChatGPT for improved decision-making in interfacility transfers for plastic surgery emergencies based on United States guidelines

**DOI:** 10.1016/j.jpra.2025.04.011

**Published:** 2025-04-26

**Authors:** Chandler Hinson, Omar Allam, Jasmine Glaspy, Trudy S. Kim, Haripriya Ayyala

**Affiliations:** a Frederick P Whiddon College of Medicine, University of South Alabama, Mobile, AL, USA; b Division of Plastic Surgery, Department of Surgery, Yale School of Medicine, New Haven, CT, USA; c Albany Medical College, Albany, NY, USA; d Division of Plastic & Reconstructive Surgery, Department of Surgery, Stanford University Medical Center, Stanford, CA, USA; e East Coast Advanced Plastic Surgery, Hoboken, NJ, USA

**Keywords:** Artificial intelligence, Plastic surgery, Triage, Emergency, Clinical decision-making

## Abstract

Plastic surgery emergencies—such as burns, facial trauma, and upper extremity injuries—often require timely specialist consultation. Yet, interfacility triage decisions are frequently made by frontline providers without direct access to plastic surgeons, leading to unnecessary transfers that burden healthcare systems and patients. Artificial intelligence (AI) platforms like ChatGPT may offer a novel way to support these decisions by aligning recommendations with established clinical guidelines. This study evaluated ChatGPT-4.0′s ability to assess the need for interfacility transfer across 60 standardized clinical scenarios representing common plastic surgery emergencies. Scenarios were based on U.S. guidelines or expert consensus and entered into ChatGPT using three different prompt formulations, or ``primers,'' yielding 180 total recommendations. These were compared against gold-standard transfer decisions. Overall, ChatGPT aligned with guideline-based decisions in 77% of cases. Performance varied by prompt: primers 1 and 3 achieved 77% alignment, while primer 2 achieved 73%. Primer 1 tended to under-refer, whereas primers 2 and 3 over-referred (*p* < 0.001). By clinical domain, alignment was highest for upper extremity trauma (83%), followed by burns (75%) and facial trauma (68%). These findings suggest ChatGPT can offer generally appropriate transfer recommendations, though its accuracy depends on both scenario type and prompt phrasing. While promising, such tools must be used with clinical oversight, given variability in performance and lack of explainability. With further validation, AI platforms like ChatGPT could support emergency triage for plastic surgery referrals, particularly in low-resource settings where timely specialist input is limited.

## Introduction

Timely plastic surgery consultation in emergency settings is vital but often hampered by geographic, institutional, or staffing limitations. In particular, rural areas and overcrowded emergency departments may lack on-site access to specialized surgical care. As a result, decisions about whether to transfer patients often fall to frontline providers with varying levels of training in plastic surgery. Over-triage—referring patients who may not require emergent intervention—is a well-documented issue that can strain hospital resources, inconvenience patients, and contribute to system-wide inefficiencies.^1^

Large language models such as ChatGPT offer new opportunities for augmenting clinical decision-making. These models, trained on large datasets of human text, have demonstrated capacity to interpret complex prompts and generate coherent, relevant responses. In plastic surgery, ChatGPT has recently been shown to perform comparably to a first year plastic surgery resident on board-style knowledge assessments.^2^ However, little is known about its real-time performance in supporting triage decisions based on clinical scenarios that commonly trigger plastic surgery referrals— facial trauma, upper extremity injuries, and burns.

**Fig. 1:**
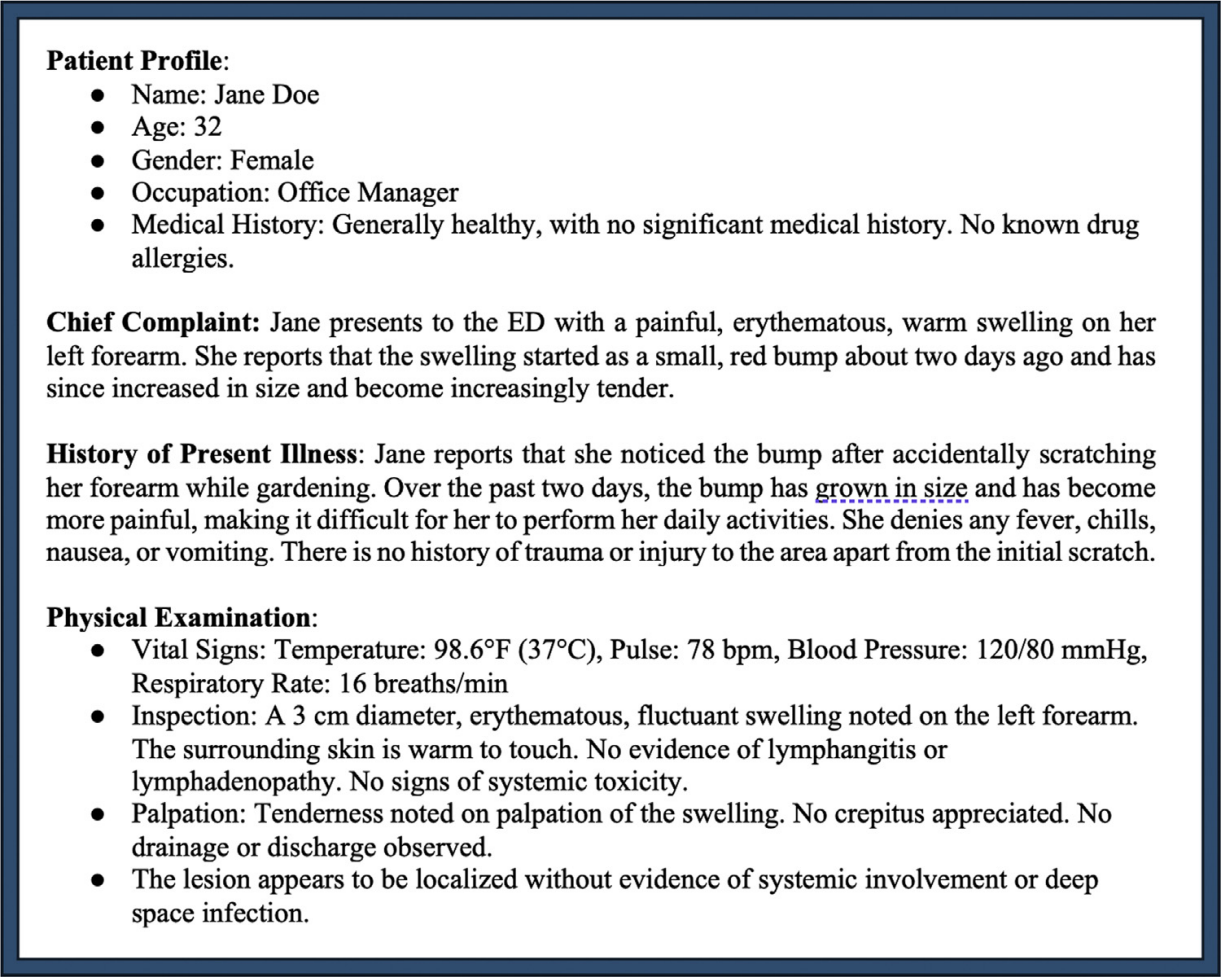
An example clinical scenario created from clinical guidelines and consensus from subject matter expert panels in the United States.

To evaluate ChatGPT’s potential as a triage adjunct, we constructed 60 clinical scenarios based on established U.S. guidelines and expert consensus documents (Fig. 1).^3^, ^4^, ^5^ The sample size was selected based on feasibility with the goal to assess a broad range of distinct scenarios across major plastic surgery emergency domains. Each scenario was entered into ChatGPT-4.0 using three different prompt formulations ("primers") to assess the effect of input phrasing on output consistency and clinical appropriateness. These primers were developed based on input from two board-certified plastic surgeons who provided the types of language and phrasing they would use when consulting ChatGPT for emergent referral decisions. The primers were:

1. “Based on the following clinical scenario, is an interfacility transfer for an emergent consult necessary?”
2. “Providing only a yes or no, does the following clinical scenario warrant an interfacility transfer for an emergent consult?”
3. “Based on the clinical diagnosis of this patient, along with not having access to a specialist at this facility, should I refer this patient for an immediate and emergent consult at another facility?”

Each scenario was entered into ChatGPT using each of the three primers, yielding 180 total outputs. These were independently reviewed and assessed for alignment with guideline-based transfer recommendations. Misalignments were categorized as either over-triage (unnecessary referral) or under-triage (failure to refer when clinically indicated). Statistical significance was evaluated using chi-square analysis.

Overall, ChatGPT's recommendations aligned with the gold-standard guideline or expert consensus in 77% of cases (Table 1). Primer phrasing influenced performance: primer 1 and primer 3 both produced 77% alignment while primer 2 produced 73% (*p* = 0.24). The nature of the errors varied significantly by primer with primer 1 significantly more likely to under-refer (93% of its misalignments), while primers 2 and 3 tended toward over-referral (88% and 100% of misalignments, respectively; *p* < 0.001). This finding is clinically meaningful, as under-triage in emergent settings could delay care, whereas over-triage may unnecessarily burden specialist services.

**Table 1:** Performance of ChatGPT in recommending interfacility transfer for plastic surgery emergencies across 60 clinical scenarios using three different prompt formulations (primers) stratified by clinical domain: upper extremity trauma, facial trauma, and burns.

Metrics include percentage alignment with guideline-based referral decisions, sensitivity, specificity, positive predictive value (PPV), and negative predictive value (NPV). “All Scenarios” reflects combined performance across all domains, while domain-specific rows reflect performance within isolated clinical contexts. The table highlights variability in ChatGPT’s outputs depending on both clinical content and prompt structure.

By clinical domain, ChatGPT aligned most accurately with referral guidelines for upper extremity trauma (83%), followed by burns (75%) and facial trauma (68%) (*p* = 0.16). These differences likely reflect variation in guideline complexity and scenario nuance. For instance, facial trauma scenarios often involve multiple systems or subtle distinctions in severity, which may challenge AI interpretation. For burn scenarios, primer 1 produced the lowest alignment (55%) compared to primers 2 and 3 (85% each, *p* = 0.041), suggesting context-specific phrasing may better guide AI outputs.

These results highlight several important themes. First, ChatGPT shows real promise as a triage support tool, particularly in under-resourced settings where plastic surgery consultation is not immediately available. Its ability to replicate clinical decision-making in structured scenarios reinforces its potential role in augmenting clinician judgment. Second, the study underscores the critical importance of prompt engineering: how the clinical question is framed can significantly impact the accuracy of AI recommendations.

However, limitations remain. A major limitation is the ``black box'' nature of ChatGPT’s responses—while its outputs are often reasonable, the model does not provide reasoning or references, making it difficult to verify or explain its conclusions. This opacity may hinder adoption among clinicians who are rightly skeptical of unvalidated tools in high-stakes environments. Moreover, its 23% rate of misalignment is nontrivial and reinforces the necessity of human oversight. AI should not replace clinical judgment but can serve as a valuable adjunct—particularly when its use is constrained by validated guidelines and implemented within well-defined parameters.

ChatGPT demonstrates a promising ability to support triage decisions for plastic surgery emergencies when clinical scenarios are clearly structured. Variability across primers and clinical domains suggests the need for further validation and the development of best practices for AI integration into emergency medicine workflows. As AI technologies evolve, continued collaboration among clinicians, researchers, and developers will be essential to ensure safety, transparency, and utility in frontline care.

## Funding

None.

## Ethical approval

Not required.

## CRediT authorship contribution statement

**Chandler Hinson:** Conceptualization, Methodology, Formal analysis, Investigation, Writing – original draft, Visualization, Project administration. **Omar Allam:** Methodology, Investigation, Formal analysis, Formal analysis, Writing – original draft, Visualization. **Jasmine Glaspy:** Validation, Formal analysis, Investigation, Writing – original draft, Visualization. **Trudy S. Kim:** Conceptualization, Formal analysis, Writing – original draft. **Haripriya Ayyala:** Methodology, Writing – original draft, Supervision, Project administration.

## Conflicts of interest

None declared.
